# Exosomal LncRNA LBX1-AS1 Derived From RBPJ Overexpressed-Macrophages Inhibits Oral Squamous Cell Carcinoma Progress *via* miR-182-5p/FOXO3

**DOI:** 10.3389/fonc.2021.605884

**Published:** 2021-03-17

**Authors:** Yilong Ai, Haigang Wei, Siyuan Wu, Zhe Tang, Xia Li, Chen Zou

**Affiliations:** Foshan Stomatological Hospital, School of Stomatology and Medicine, Foshan University, Foshan, China

**Keywords:** OSCC, macrophage, exosomal lncRNA, RBPJ, FOXO3

## Abstract

**Objectives:**

Macrophage-derived exosomes (Mφ-Exos) are involved in tumor onset, progression, and metastasis, but their regulation in oral squamous cell carcinoma (OSCC) is not fully understood. *RBPJ* is implicated in macrophage activation and plasticity. In this study, we assessed the role of Mφ-Exos with *RBPJ* overexpression (*RBPJ-OE* Mφ-Exos) in OSCC.

**Materials and Methods:**

The long non-coding RNA (lncRNA) profiles in *RBPJ-OE* Mφ-Exos and THP-1-like macrophages (WT Mφ)-Exos were evaluated using lncRNA microarray. Then the functions of Mφ-Exo-lncRNA in OSCC cells were assessed *via* CCK-8, EdU, and Transwell invasion assays. Besides, luciferase reporter assay, RNA immunoprecipitation, and Pearson’s correlation analysis were adopted to confirm interactions. Ultimately, a nude mouse model of xenografts was used to further analyze the function of Mφ-Exo-lncRNAs *in vivo*.

**Results:**

It was uncovered that lncRNA *LBX1-AS1* was upregulated in *RBPJ-OE* Mφ-Exos relative to that in WT Mφ-Exos. *RBPJ-OE* Mφ-Exos and *LBX1-AS1* overexpression inhibited OSCC cells to proliferate and invade. Meanwhile, *LBX1-AS1* knockdown boosted the tumor to grow *in vivo*. The effects of *RBPJ-OE* Mφ-Exos on OSCC cells can be reversed by the *LBX1-AS1* knockdown. Additionally, mechanistic investigations revealed that *LBX1-AS1* acted as a competing endogenous RNA of miR-182-5p to regulate the expression of *FOXO3*.

**Conclusion:**

Exo-*LBX1-AS1* secreted from *RBPJ-OE* Mφ inhibits tumor progression through the *LBX1-AS1*/miR-182-5p/*FOXO3* pathway, and *LBX1-AS1* is probably a diagnostic biomarker and potential target for OSCC therapy.

## Introduction

Oral squamous cell carcinoma (OSCC) is one of the most aggressive head and neck cancers and has a poor survival rate (1). Although multiple therapeutic strategies can be administered clinically to treat OSCC, the overall 5-year survival rate after diagnosis remains less than 50%, mainly owing to cancer metastasis to lymph nodes and distant sites (2, 3). Therefore, a better understanding of the molecular mechanisms underlying OSCC metastasis is essential to develop novel therapies against OSCC.

Increasing evidence unveils that exosomes (Exos) mediate the interactions between macrophages and cancer cells (4–6). M2 macrophage-derived exosomes facilitate hepatocarcinoma metastasis by transferring α β integrin to tumor cells (7), and M2 bone marrow-derived macrophage-derived exosomes shuffle microRNA-21 to accelerate immune escape of glioma by modulating PEG3 (8). Besides, downregulated lncRNA SBF2-AS1 in M2 macrophage-derived exosomes elevates miR-122-5p to restrict XIAP, thereby limiting pancreatic cancer development (9).

Long non-coding RNAs (lncRNAs) are a class of transcripts longer than 200 nucleotides with no protein-coding capacity and are poorly conserved (10). LncRNAs have also been found in Exos (11), and they are thought to modulate the expression of genes and miRNAs (12). Exosome-Transmitted lncRNAs can regulate growth of cancers (13, 14). Recent studies have unveiled the involvement of lncRNAs in OSCC progression by competitive sponging miRNAs (15, 16). For instance, lncRNA RC3H2 facilitates cell proliferation by targeting microRNA-101-3p/EZH2 axis in OSCC (17). However, whether Mφ-Exo-lncRNAs can regulate the progression of OSCC is unclear.

The Notch pathway is involved in several cancers progression (18, 19), and it is also believed to be responsible for the activation and differentiation of macrophages (20, 21). The recombination signal binding protein for immunoglobulin kappa J region (*RBPJ*) is often used as a marker for the activation of Notch signaling (22). Upon ligand binding, the Notch intracellular domain translocates into the nucleus and forms a complex with the transcription factor RBPJ to activate expression of Notch target genes (22). Loss of the Notch effector *RBPJ* promotes tumorigenesis (23). Moreover, Notch-*RBPJ* signal transduction regulates the transcription factor IRF8 to facilitate inflammatory macrophage polarization (24).

In the current research, we probed the impacts of Mφ-Exos overexpressing *RBPJ* (*RBPJ-OE* Mφ-Exos) on OSCC cell proliferation and invasion and compared them with Exos from THP-1-like macrophages (WT Mφ-Exos) (25). To further understand the regulatory mechanism of *RBPJ-OE* Mφ-Exos in OSCC, we also determined the differentially regulated lncRNAs when *RBPJ* was upregulated in Mφ-Exos. In addition, we identified the miRNA binding partners of the lncRNA upregulated in *RBPJ-OE* Mφ-Exos and their targets. The aim of this study was to further understand the mechanisms of macrophage-derived exosomes-lncRNA, and to identify diagnostic biomarkers and potential therapeutic targets.

## Materials and Methods

### Cell Culture and Clinical Specimens

Human monocytic cell line THP-1 and OSCC cell lines (SCC-4 and CAL-27) were purchased from the Institute of Biochemistry and Cell Biology of the Chinese Academy of Sciences (Shanghai, China). THP-1 cells were cultured in RPMI-1640 medium provided by Gibco (Shanghai, China), and OSCC cells were cultured in Dulbecco’s Modified Eagle medium (DMEM, Gibco, China) with 10% heat-inactivated fetal bovine serum (FBS) from Thermo Fisher Scientific (Shanghai, China), 100 U/ml penicillin, and 100 µg/ml streptomycin from HyClone Laboratories (Beijing, China) at 37°C in a moist incubator with 5% CO_2_ and used in the exponential growth phase.

Forty paired OSCC tissues and para-tumor tissues were obtained from patients receiving surgery at Foshan Stomatological Hospital between 2016 and 2019. They were diagnosed by histopathology and received no treatment prior to the operation. Besides, all participants signed informed consent in written form before the research. This research gained the approval of the Ethics Committees of Foshan Stomatological Hospital, School of Stomatology and Medicine, Foshan University (FSU2016033), and was conducted *as per* the Helsinki Declaration.

### Isolation of Exos Derived From THP-1 Mφ Cells With or Without the Overexpression of *RBPJ*

To obtain WT Mφ and *RBPJ-OE* Mφ, THP-1 cells underwent transfection with the pCMV6 empty vector or pCMV6 overexpressing *RBPJ* (OriGene, Rockville, MD, USA) and seeded at 1 × 10^6^ cells/well in a six-well culture plate. Gradient centrifugation was utilized for Exo isolation from the cell culture medium. Specifically, the medium underwent 30-min centrifugation at 3,000 × g for removal of cells and cellular debris. Subsequent to collection of the supernatant, the medium underwent 30-min centrifugation again at 10,000 × g to discard larger microvesicles. Finally, 70-min Exo isolation from the supernatant was implemented at 110,000 × g and 4°C, and the Exos were reserved in phosphate buffered saline (PBS) at −80°C.

### Transmission Electron Microscopy Assay

Exos for transmission electron microscopy (TEM) were prepared as mentioned above. Briefly, Exos were first fixed in 2.5% glutaraldehyde (pH 7.2) at 4°C, then washed in PBS, embedded in 10% gelatin, and fixed in 1% osmium tetroxide for 60 min at indoor temperature. Next, the embedded Exos were cut into 1 mm-thick blocks and dehydrated with gradient alcohol. The alcohol was then replaced with gradient mixture of Quetol-812 epoxy resin and propylene oxide. Afterwards, samples were embedded in Quetol-812 epoxy resin, polymerized at a temperature gradient, and cut into ultrathin sections using a Leica UC6 ultramicrotome. Finally, subsequent to dying by uranyl acetate and lead citrate, a transmission electron microscope was utilized for section observation.

### Microarray Analysis

The isolation and quantification of the total RNAs were independently implemented using Trizol reagent provided by Life Technologies (Shanghai, China) and NanoDrop ND-1000. The enriched lncRNAs were then amplified and labeled fluorescently using the Quick Amp Labeling Kit (Agilent Technologies) *as per* the guideline of the manufacturer. In the meantime, we hybridize the labeled cRNAs onto an Arraystar Human lncRNA Array (8× 60K, version 2.0). Subsequent to rinsing and scanning of slides using an Agilent Scanner G2505B, the obtained images were assessed *via* Agilent Feature Extraction software (version 10.7.3.1). Quantile normalization and subsequent data processing were performed using GeneSpring GX software, version 11.5.1 (Agilent Technologies). Volcano plot filtering was used to identify the lncRNAs with statistically significant differences.

### RNA Extraction and Quantitative Real-Time PCR (qRT-PCR)

The reverse transcription of mRNAs and lncRNAs into cDNAs was implemented using a reverse transcription kit from Takara (Beijing, China). Next, cDNAs were subjected to RT-PCR on a Quantstudio™ DX system (Applied Biosystems, Singapore) under the following conditions: denaturation at 95°C for 30 s and (denaturation at 95°C for 5 s, at 60°C for 10 s, and at 72°C for 30 s) ×40 cycles. Afterwards, we utilized 2^-ΔCT^ or 2^-ΔΔCT^ to quantify mRNAs and lncRNAs by normalizing to GAPDH (26) and to determine the relative expression subsequent to the normalization of miRNA expression to small nuclear U6. Each experiment was separately performed in triplicate. All PCR primers were listed in **Table 1**.

**Table 1:** Sequences of primers for qRT-PCR and siRNA related sequence.

Name		Sequence
LBX1-AS1	Forward	5’- CAGGCGTTCCTTTCTTTCTG-3’
	Reverse	5’- AGGACAGACGCTTGAGGAAA-3’
FOXO3	Forward	5’- CGGACAAACGGCTCACTCT-3’
	Reverse	5’- GGACCCGCATGAATCGACTAT-3’
GAPDH	Forward	5’-GGCTGTTGTCATACTTCTCATGG-3’
	Reverse	5’-GGATCTCGCTCCTGGAAGATG-3’
U6	Forward	5’-CTCGCTTCGGCAGCACA-3’
	Reverse	5’-AACGCTTCACGAATTTGCGT-3’
miR-182-5p	Forward	5’- ACACTCCAGCTGGGTTTGGCAATGGTAGAACT-3’
	Reverse	5’- CTCAACTGGTGTCGTGGAGTCGGCAATTCAGTTGAGAGTGTGAG-3’
LBX1-AS1 siRNA	Sense	5’- GGGGCGAGGAGGCGAGGGCUU-3’
	Antisense	5’- GCCCUCGCCUCCUCGCCCCUU-3’
miR-182-5p mimics	Sense	5’- UUUGGCAAUGGUAGAACUCACACU -3’
	Antisense	5’- UGUGAGUUCUACCAUUGCCAAAUU-3’
miR-182-5p inhibitor	Sense	5’- AGUGUGAGUUCUACCAUUGCCAAA-3’

### Cell Transfection

*LBX1-AS1* overexpression plasmid (p-lncRNA) and its negative control pcDNA3.1, small interfering RNAs (siRNAs) targeting *LBX1-AS1* and non-specific negative control oligos (si-NC), miR-182-5p mimics, inhibitor, and the negative control (NC), and the shRNAs were synthetized by GeneChem (Shanghai, China). Detailed sequences were depicted in **Table 1**. SCC-4 and CAL-27 cell lines underwent inoculation in six-well plates at 24 h prior to transfection with pcDNA3.1, p-lncRNA, si-NC, si-lncRNA, and miR-182-5p mimics or inhibitor under 50–60% cell confluence using Lipofectamine 3000 (Invitrogen) as per the guideline of the manufacturer. Later, the effects of knockdown or overexpression were examined by qRT-PCR using the RNAs that were extracted after 48-h transfection. For Exo treatment, OSCC cells were cultured in medium containing 5 μg/ml Exos from WT Mφ, *RBPJ-OE* Mφ or si-lncRNA and *RBPJ-OE* Mφ.

### Cell Proliferation Assays

Approximately 1.0 × 10^4^ transfected SCC-4 and CAL-27 cells were cultured in 96-well plates, and then underwent 1-h incubation with CCK-8 reagent (Beyotime, Shanghai, China). The absorbance at 450 nm was recorded using an Infinite M200 multimode microplate reader (Tecan, Shanghai, China).

After approximately 48-h transfection, the 5-ethynyl-2’-deoxyuridine (EdU) assay kit provided by Ribo (Guangzhou, China) was utilized to examine the proliferation of SCC-4 and CAL-27 cells. Specifically, cells were grown in culture medium containing EdU (Invitrogen) solution (1:1,000). At the proliferative stage, the cells were labeled with EdU for 2 h, followed by rinsing with PBS (0.5 g/ml) thrice. Subsequently, the cells were stained by 4′,6-diamidino-2-phenylindole (DAPI) from Invitrogen for 10 min at indoor temperature in the dark and underwent PBS rinsing more than twice. Ultimately, assessment of the stained cells was implemented *via* the FACSCalibur DxP flow cytometer (BD Biosciences, Shanghai, China).

### Cell Invasion Assays

Cell invasion was assessed in the Matrigel assay using the 24-well invasion chamber system equipped with polycarbonic membranes (diameter 6.5 mm, pore size 8 μm) from BD Biosciences (Santa Clara, CA, USA). Subsequent to incubation and dying, a microscope was adopted to quantify cells co-cultured with Exos and migrating through the membranes in four fields that were randomly chosen. Each assay was repeated at least three times with triplicate samples each time.

### Luciferase Reporter Assay

Sequences of WT or MUT *LBX1-AS1* or the full length of the 3′-UTR of *FOXO3* with WT or MUT putative binding sites were interposed into the pmir-GLO vector from Promega Corp. (Beijing, China). 293T cells seeded into 24-well plates underwent co-transfection with 50 nM miR-182-5p mimics or a NC and 80 ng WT or MUT plasmids using Lipofectamine 2000 (Invitrogen)and the 80 ng of plasmids were later added with 5 ng of pRL-SV40. Lastly, luciferase intensity was determined using the Dual-Luciferase Reporter Assay Kit from Promega (Beijing, China) and a microplate reader.

### RNA Binding Protein Immunoprecipitation (RIP) Assay

We carried out the RIP assay using a Magna RIP Kit from Millipore (Hongkong, China) *as per* the guideline of the manufacturer. Specifically, cells (1 × 10^7^) were lysed with the lysis buffer provided in the kit and the lysate was separately put into two tubes [one with anti-Argonaute2 (AGO2) antibody and the other with a non-specific anti-IgG antibody (Millipore)]. The cell lysates were incubated nightlong at 4°C, and then incubated with magnetic beads for a further hour. Proteinase K was then added for sample incubation at 55°C for another hour. In the end, RNA extraction reagent (Solarbio, Beijing, China) was used to obtain the RNAs, and specific genes were detected and measured using qRT-PCR.

### Western Blot Analysis

Cell lysis was performed in RIPA buffer (Beyotime, Nantong, China) containing protease and phosphatase inhibitors (Beyotime). A BCA Protein Assay kit (Beyotime) was utilized to identify protein concentration, and the samples (40 µg proteins per lane) underwent SDS-PAGE with 10% gel for separation. Next, proteins were electrotransferred onto a PVDF membrane (Beyotime) that was sealed by 5% BSA (Beyotime) for 1 h at indoor temperature. Later, we incubated the membrane with primary antibodies against TSG101 (1:1,000, ab125011, Abcam, Shanghai, China), CD63 (1:1,000, ab217345, Abcam, Shanghai, China), *FOXO3* (1:1,000, ab23683, Abcam, Shanghai, China), and GAPDH (1:1,000, ab8245, Abcam, Shanghai, China) at 4°C nightlong, and subsequently with secondary antibodies coupled to HRP (Beyotime, Nantong, China) at indoor temperature for 1 h. Immobilon ECL substrate (Millipore) was used to generate signals, which were detected using the Optimax X-ray Film Processor provided by Protec (Shanghai, China).

### Xenograft Nude Mouse Model

Six-week-old adult male BALB/C nude mice (n = 3/group) were commercially provided by Shanghai SLAC Laboratory Animal Co., Ltd. (Shanghai, China) and reserved in a SPF environment with a LD (12:12) cycle. All animal studies obtained the approval of the Institutional Animal Care and Use Committee of Foshan Stomatological Hospital, School of Stomatology and Medicine, Foshan University, and implemented in line with institutional and national guidelines. SCC-4 cells undergoing stable sh-NC or sh-lncRNA transfection, or WT Mφ-Exo, *RBPJ-OE* Mφ-Exo or *RBPJ-OE* Mφ-Exo-sh-lncRNA (5 μg/ml) pretreatment were hypodermically injected into the nude mice (1 × 10^6^ cells per mouse) on the right upper back. Later, we utilized a caliper to determine the growth of tumor every 7 days for 35 days, and calculate its volume based on the formula: volume = (length × width^2^)/2. Five weeks later, we intraperitoneally injected overdose pentobarbital (>120 mg/kg body weight) to kill all the mice so that they were unable to spontaneously breath. Afterwards, the xenograft tumor tissues were sampled for subsequent analyses.

### Statistical Analysis

GraphPad Prism 6.0 software provided by GraphPad Inc. (San Diego, CA, USA) was utilized to statistically evaluate data. Experimental results were presented as mean ± standard deviation (SD). The statistically significant differences between tumor tissues and para-tumor tissues were determined using paired Student’s t-test. Besides, the statistically significant differences between other two groups were detected using Mann-Whitney U-test or unpaired Student’s t-test in light of conditions. Furthermore, the comparisons among different groups (multigroup comparisons) were implemented by one-way ANOVA and the *post hoc* Bonferroni test. Lastly, Pearson’s correlation coefficient was determined to test associations among *LBX1-AS1*, miR-182-5p, and *FOXO3*. P < 0.05 signified statistically significant differences.

## Results

### Mφ-Exos Overexpressing *RBPJ* Inhibit Proliferation and Invasion of OSCC Cells

**Figure 1:**
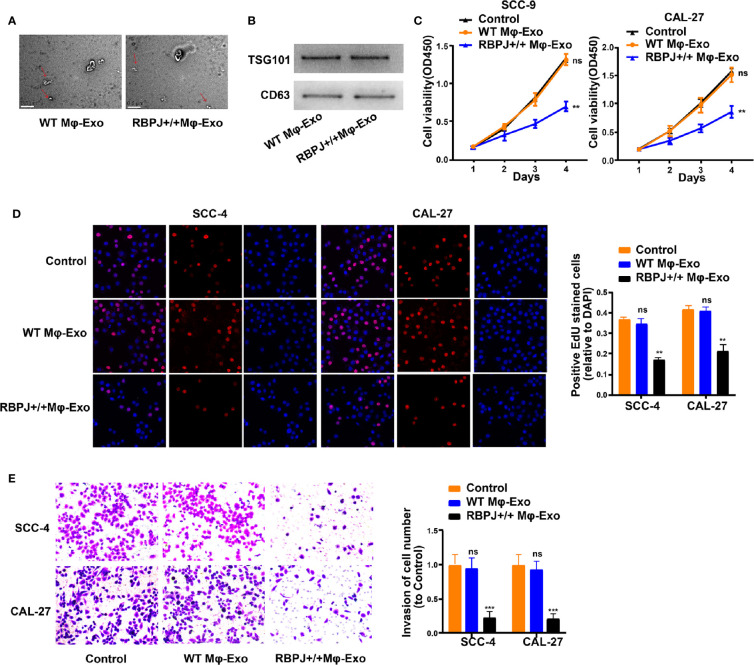
Exosomes derived from macrophages overexpressing RBPJ inhibit proliferation and invasion of OSCC cells. **(A)** Exosomes isolated from WT THP-1 derived macrophages (WT Mφ-Exos) and RBPJ-overexpressed macrophages (RBPJ-OE Mφ-Exos) imaged by transmission electron microscopy (TEM). Scale bar = 0.5 μm. **(B)** Levels of exosome markers CD63 and TSG101 in WT or RBPJ-OE Mφ-Exos were determined by Western blotting. **(C, D)** Cell proliferation in OSCC cell lines SCC-4 and CAL-27 treated with WT Mφ-Exos, RBPJ-OE Mφ-Exos, or negative control was assessed by CCK-8 **(C)** and EdU assay **(D)**. **(E)** Transwell invasion assay is performed to indicate cell invasion. All experiments were performed three times. **P < 0.01 and ***P < 0.001 for statistical difference, ns: no significance.

WT Mφ-Exos and *RBPJ-OE* Mφ-Exos were isolated by ultracentrifugation and characterized by TEM (**Figure 1A**). To further confirm the identity of the Exos, the expression levels of CD63 and TSG101 (Exo markers) were evaluated. Western blot assessment showed that the isolated Exos were enriched with CD63 and TSG101 (**Figure 1B**). These data indicate the successful isolation of Exos from WT Mφ-Exos and *RBPJ-OE* Mφ-Exos.

To further investigate the effects of these two groups of Exos on the proliferation of OSCC cells, we cocultured SCC-4 or CAL-27 cells with Exos for 4 days and measured cell proliferation through CCK-8 and EdU experiments. As demonstrated in **Figures 1C–D**, the presence of *RBPJ-OE* Mφ-Exos significantly curbed SCC-4 and CAL-27 cells to proliferate when compared with WT Mφ-Exos or negative control groups. In addition, Transwell invasion assays indicated that *RBPJ-OE* Mφ-Exos were able to inhibit invasion of SCC-4 and CAL-27 cells (**Figure 1E**). Overall, these results confirm that the overexpression of *RBPJ* in Exos can suppress OSCC cells to proliferate and invade.

### Expression Profiles of lncRNAs in *RBPJ-OE* Mφ-Exos

**Figure 2:**
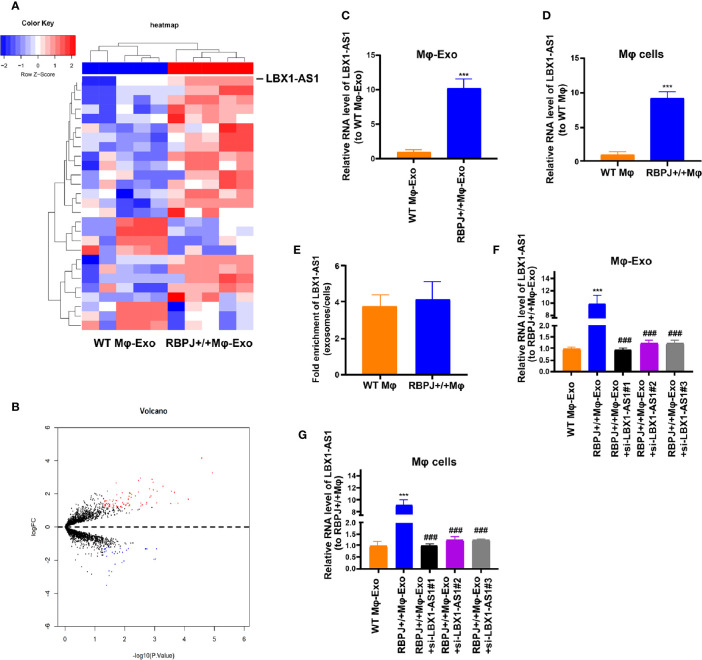
LBX1-AS1 expression profiles in exosomes derived from RBPJ-overexpressed macrophages. **(A)** Cluster heatmap showing 27 aberrantly expressed LBX1-AS1s, including 24 upregulated and 3 downregulated LBX1-AS1s in exosomes derived from RBPJ-overexpressed macrophages compared to the controls. The red color represents high expression, whereas the blue color represents low expression. **(B)** Volcano map. **(C, D)** The relative expression of LBX1-AS1 in Mφ-Exos **(C)** and Mφ cells **(D)** was validated by qRT-PCR. **(E)** The fold change of LBX1-AS1 expression between the exosomes and their corresponding producer cells. **(F, G)** The qRT-PCR assay indicated the difference in the LBX1-AS1 expression in RBPJ-overexpressed Mφ cells transfected with or without LBX1-AS1 siRNA **(F)**, as well as in exosomes from those cells **(G)**. ***P < 0.001 *vs* WT Mφ-Exos, ^###^P < 0.001 *vs* RBPJ-OE Mφ-Exos.

We wonder whether *RBPJ-OE* Mφ-Exos could influence the expression of *RBPJ* in OSCC cells. We performed qRT-PCR to detect the expression of *RBPJ* in the WT Mφ, *RBPJ*-OE Mφ, WT Mφ-Exos, *RBPJ*-OE Mφ-Exos, WT Mφ-Exos treated SCC-4/CAL-27 cells, and *RBPJ*-OE Mφ-Exos treated SCC-4/CAL-27 cells. Our results showed that *RBPJ* mRNA is highly expressed in *RBPJ*-OE Mφ (**Supplementary Figure 1A**). Nevertheless, there is no significant difference of *RBPJ* level between WT Mφ-Exos treated SCC-4/CAL-27 cells and *RBPJ*-OE Mφ-Exos treated SCC-4/CAL-27 cells (**Supplementary Figure 1B**). As the PCR amplification cycles of *RBPJ* in WT Mφ-Exos and *RBPJ*-OE Mφ-Exos are higher than 40, we can’t detect the expression level of it in the exosomes. This result means that *RBPJ* could not be enriched in macrophage exosomes. We wonder whether there are different expressions of lncRNAs between WT Mφ-Exos and *RBPJ-OE* Mφ-Exos. The lncRNA profiles in *RBPJ-OE* Mφ-Exos and WT Mφ-Exos were evaluated using a lncRNA microarray technique. Twenty-seven lncRNAs were differentially expressed (P < 0.05 and log2FC > 2.0 or < −2.0) in *RBPJ-OE* Mφ-Exos and the controls (**Figures 2A, B**). We have uploaded the data of this lncRNA profiles on ArrayExpress with the accession E-MTAB-9989. Among them, 24 lncRNAs dramatically rose up and 3 lncRNAs evidently declined. *LBX1-AS1* with the most obvious rising trend was selected and validated by qRT-PCR in *RBPJ-OE* Mφ-Exos and WT Mφ-Exos (**Figure 2C**). In the meantime, it was unveiled that *LBX1-AS1* was expressed in the *RBPJ-OE* Mφ at a notably higher level relative to that in the WT Mφ cells (**Figure 2D**). Compared with those in the producer cells, the levels of *LBX1-AS1* are enriched by approximately 4 folds in the *RBPJ-OE* Mφ-Exos (**Figure 2E**).

### Mφ-Exo-*LBX1-AS1* Inhibits OSCC Cells to Proliferate and Invade

**Figure 3:**
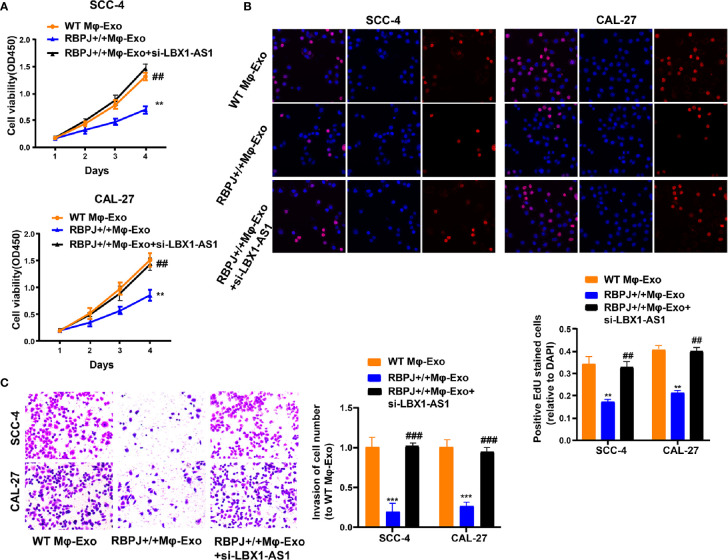
Mφ-Exo-LBX1-AS1 inhibits proliferation and invasion of OSCC cells. To remove LBX1-AS1 from exosomes, siRNA of LBX1-AS1 was transfected into THP-1 cells and Mφ-Exos were collected at 48 h post-transfection (RBPJ-OE Mφ-Exo-si-LBX1-AS1). OSCC cell lines SCC-4 and CAL-27 were cocultured with WT Mφ-Exos, RBPJ-OE Mφ-Exos, or RBPJ-OE Mφ-Exo-si-LBX1-AS1. **(A, B)** Cell proliferation in OSCC cell lines SCC-4 and CAL-27 was assessed by CCK-8 assay **(A)** and EdU assay **(B)**. **(C)** Cell invasion in OSCC cell lines SCC-4 and CAL-27 was assessed by Transwell assay. All experiments were performed three times. **, ^##^P < 0.01 and ***, ^###^P < 0.001 as indicated. **/*** vs. WT Mφ-Exos, ##/### vs. RBPJ^+/+^ Mφ-Exos.

Since the *LBX1-AS1* level was the highest in *RBPJ-OE* Mφ-Exos, to remove its expression from Exos, the siRNA of *LBX1-AS1* was transfected into Mφ cells for 48 h, after which Exos were collected (**Figures 2F–G**). Next, OSCC cell proliferation and invasion were investigated by coculturing cells with Mφ-Exos. The inhibitory effects of *RBPJ-OE* Mφ-Exos on the proliferation and invasion of OSCC cells (SCC-4 and CAL-27) were eliminated when *LBX1-AS1* was knocked down in Exos (**Figures 3A–C**). This would be expected if there was an association between *RBPJ* and *LBX1-AS1*.

**Figure 4:**
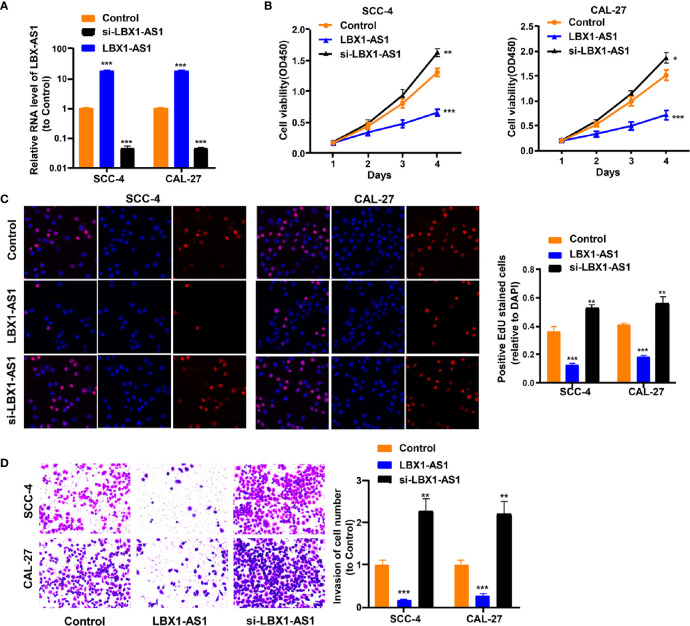
LBX1-AS1 inhibits proliferation and invasion of OSCC cells. **(A, B)** SCC-4 and CAL-27 cells were transfected with LBX1-AS1 overexpression plasmids, siRNA and controls. **(B, C)** Cell proliferation in OSCC cell lines SCC-4 and CAL-27 was assessed by CCK-8 assay **(B)** and EdU assay **(C)**. **(D)** Cell invasion of OSCC cell lines SCC-4 and CAL-27 was assessed by Transwell invasion assay. All experiments were performed three times. *P < 0.05, **P < 0.01, and ***P < 0.001 for statistical differences.

To continuously figure out the biological role of *LBX1-AS1* in OSCC cells, SCC-4 and CAL-27 cells underwent transfection with a *LBX1-AS1* overexpression vector or siRNA#1 (**Figure 4A**). The results unveiled that *LBX1-AS1* overexpression significantly inhibited OSCC cells to proliferate and invade and *LBX1-AS1* downregulation significantly promoted it (**Figures 4B–D**). As with the overexpression of *RBPJ*, the overexpression of *LBX1-AS1* inhibited proliferation and invasion of OSCC cells.

### LBX1-AS1 Interacts With miR-182-5p

**Figure 5:**
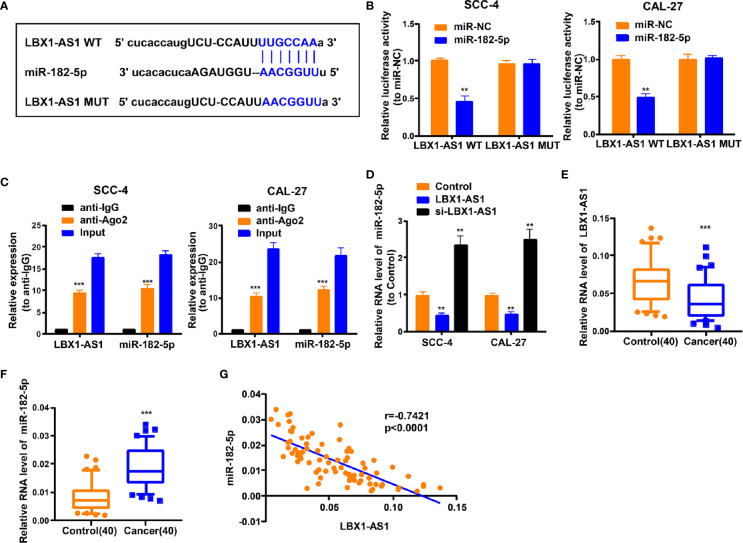
LBX1-AS1 interacts with miR-182-5p. **(A)** Putative complementary sites within miR-182-5p and LBX1-AS1 were predicted by bioinformatics analysis. **(B)** Dual-luciferase reporter assays demonstrate that miR-182-5p is a direct target of LBX1-AS1 in OSCC cells. **(C)** The Ago2 RIP showed that Ago2 significantly enriched LBX1-AS1 and miR-182-5p. **(D)** The level of miR-182-5p was determined by qRT-PCR in SCC-4 and CAL-27 cells after transfection. **(E)** The expression level of LBX1-AS1 in 40 OSCC tissues and matched para-carcinoma normal tissues was determined by qRT-PCR. **(F)** The expression level of miR-182-5p in the above tissues was determined by qRT-PCR. **(G)** The expression levels of miR-182-5p are negatively correlated with LBX1-AS1 in OSCC tissues. **P < 0.01 and ***P < 0.001 for statistical differences.

For discovering more about the specific regulation of *LBX1-AS1*, we performed bioinformatics prediction (starBase). Bioinformatics analysis predicted that *LBX1-AS1* and miR-182-5p possessed complementary binding sites (**Figure 5A**). Then we carried out a dual-luciferase experiment in 293T cells to confirm this interaction by mutating the predicted binding site in *LBX1-AS1*. It was unveiled that the luciferase activity was reduced only in presence of WT *LBX1-AS1* and miR-182-5p mimics in OSCC cells (**Figure 5B**), which was further validated using an Ago2 RIP assay. MiRNA is a component of the RNA-induced silencing complex (RISC) containing Ago2. Ago2 is required for miRNA-mediated gene silencing. In this study, we analyzed if LBX1-AS1 and miR-182-5p constitute the same RISC and performed RIP assay in OSCC cells. It is shown that LBX1-AS1 and miR-182-5p were enriched in the immunoprecipitation from anti-Ago2 group than IgG control (**Figure 5C**). Moreover, the level of miR-182-5p was prominently lower in cells overexpressing *LBX1-AS1* and higher in cells with *LBX1-AS1* silenced (**Figure 5D**), indicating that miR-182-5p expression was strongly influenced by the level of *LBX1-AS1*. Besides, levels of *LBX1-AS1* and miR-182-5p were also analyzed in OSCC and matched para-carcinoma tissues, and the results further substantiated that OSCC tissues exhibited a lower *LBX1-AS1* level (**Figure 5E**) and a higher miR-182-5p level (**Figure 5F**). Besides, Pearson’s analysis confirmed a negative interrelation between *LBX1-AS1* and miR-182-5p (**Figure 5G**). This indicates that *LBX1-AS1* may compete to interact with miR-182-5p and prevent it from regulating other pathways.

### *LBX1-AS1* Represses OSCC Cells to Proliferate and Invade *via* the miR-182-5p/*FOXO3* Pathway

**Figure 6:**
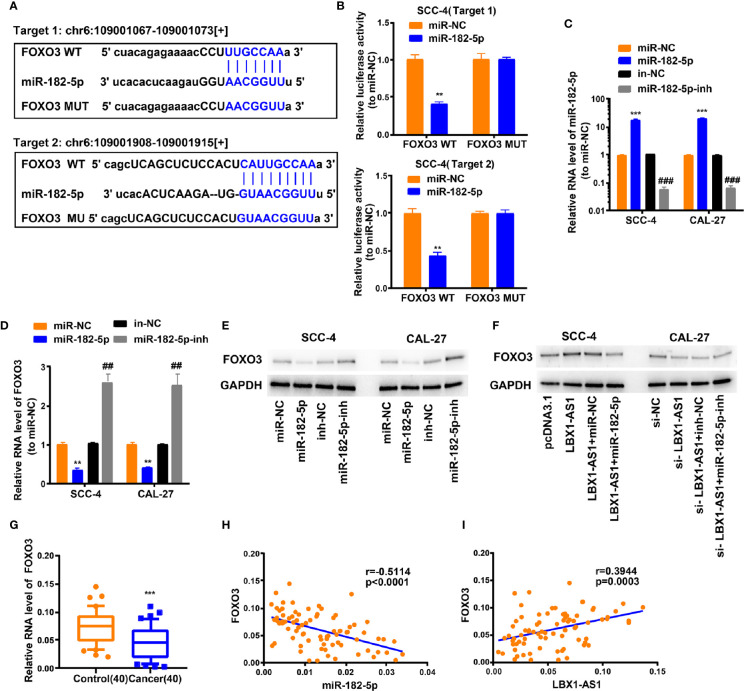
LBX1-AS1/miR-182-5p axis is critical for FOXO3 expression. **(A)** Bioinformatics analysis revealed the predicted binding sites between FOXO3 and miR-182-5p. **(B)** Luciferase reporter assay demonstrated miR-182-5p mimics significantly decreased the luciferase activity of FOXO3-WT in SCC-4 cells. **(C)** The transfection efficiency of miR-182-5p mimics and inhibitor in SCC-4 and CAL-27 cells. **(D, E)** The mRNA **(D)** and protein **(E)** level of FOXO3 was detected through qRT-PCR and western blotting after transfection with miR-182-5p mimics and inhibitor in SCC-4 and CAL-27 cells. **(F)** LBX1-AS1 overexpression upregulated FOXO3 and siRNA downregulated FOXO3, this effect can be reversed by co-transfection with miR-182-5p mimics or miR-182-5p inhibitors (miR-182-5p-inh) in SCC-4 or CAL-27 cells respectively. **(G)** The expression levels of FOXO3 in 40 OSCC tissues and matched para-carcinoma normal tissues was determined by qRT-PCR. **(H)** Expression levels of FOXO3 negatively correlated with miR-182-5p in OSCC tissues. **(I)** Expression levels of FOXO3 positively correlated with LBX1-AS1 in OSCC tissues. **, ^##^P < 0.01 and ***, ^###^P < 0.001 as indicated. * vs. miR-NC; Control, # vs. in-NC.

**Figure 7:**
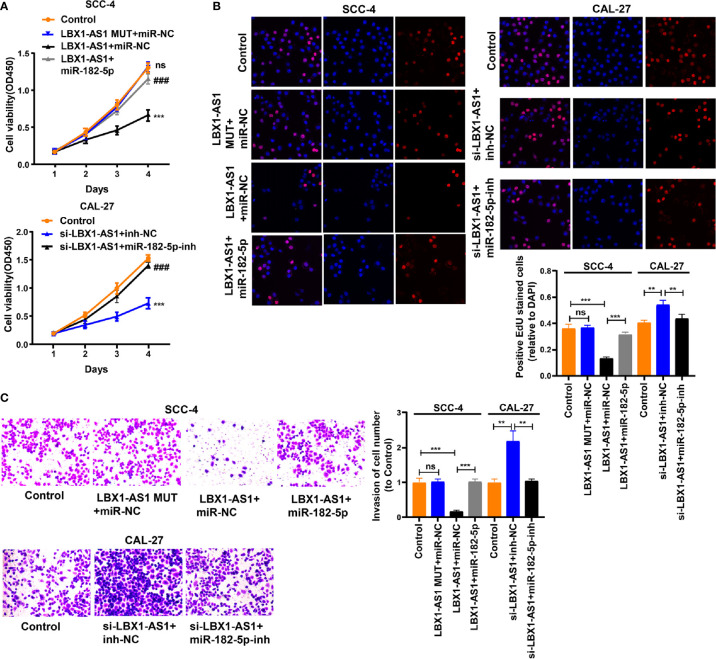
LBX1-AS1 inhibits proliferation and invasion of OSCC cells by interacting with miR-182-5p and upregulating FOXO3 expression. **(A, B)** Cell proliferation in OSCC cell lines SCC-4 and CAL-27 was assessed using CCK-8 assay **(A)** and EdU assay **(B)**. **(C)** Cell invasion of OSCC cell lines SCC-4 and CAL-27 was assessed by Transwell invasion assay. All experiments were performed three times. **P < 0.01 and ***, ^###^P < 0.001 as indicated, ns, no significance. **/*** and ns *vs.* Control, ### *vs.* p-LBX1-AS1+miR-182-5p NC.

We next probed the potential binding sites of miR-182-5p. Target prediction and assessment were implemented using starBase (http://starbase.sysu.edu.cn) and miRDB (http://mirdb.org), which identified that miR-182-5p probably interacts with *FOXO3*, a tumor suppressor gene implicated in several cancers (27–29). Recent study also pointed out that miR-182-5p promotes hepatocellular carcinoma progression by repressing FOXO3a (30). Later, we mutated two potential miR-182-5p target sites in *FOXO3* (**Figure 6A**) and performed a luciferase reporter experiment, which ascertained that miR-182-5p overexpression in SCC-4 cells dramatically weakened the luciferase activity of *FOXO3* at both target sites (**Figure 6B**). Thereafter, we examined the transfection efficiency of miR-182-5p mimics and inhibitor (**Figure 6C**). The mRNA and protein levels of *FOXO3* dropped down in SCC-4 and CAL-27 cells overexpressing miR-182-5p but rose up in OSCC cells undergoing miR-182-5p inhibitor transfection (**Figures 6D–E**). Overexpression of *LBX1-AS1* upregulated *FOXO3* whereas miR-182-5p mimics transfection reversed it in SCC-4 cells (**Figure 6F**). Besides, downregulation of *LBX1-AS1* inhibited *FOXO3* whereas miR-182-5p inhibitor transfection reversed it in CAL-27 cells (**Figure 6F**). Relative to the matched paracarcinoma tissues, *FOXO3* was expressed at a lower level in OSCC tissues (**Figure 6G**). In OSCC tissues, *FOXO3* was negatively correlated with miR-182-5p, but positively correlated with *LBX1-AS1* expression (**Figures 6H–I**). Further, *LBX1-AS1* overexpression inhibited cells to proliferate and invade whereas miR-182-5p mimics transfection reversed it in SCC-4 and CAL-27 cells (**Figures 7A–C**). Nevertheless, LBX1-AS1 mutant plasmid can’t inhibit the cell proliferation and invasion of OSCC cells (**Figures 7A–C**). It can be assumed that *LBX1-AS1* inhibits proliferation and invasion in OSCC cells by interacting with miR-182-5p and upregulating *FOXO3* expression.

### *RBPJ-OE* Mφ-Exos Inhibits Tumor Growth Through the *LBX1-AS1*/miR-182-5p/*FOXO3* Pathway *In Vivo*

**Figure 8:**
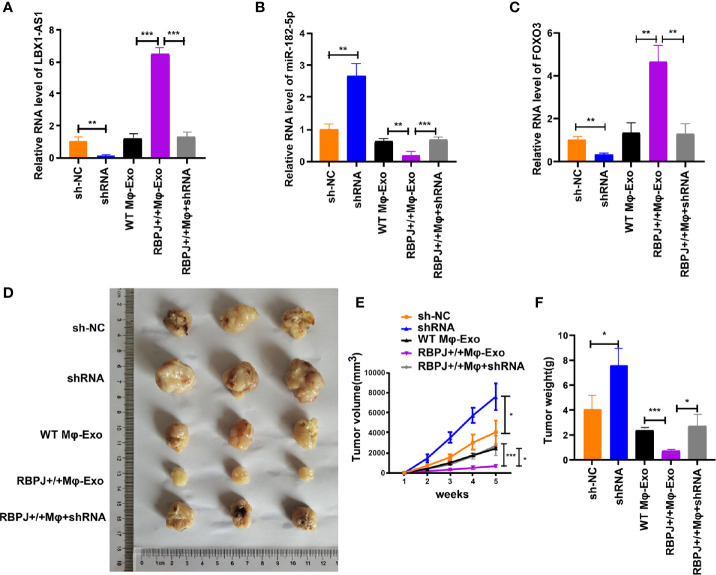
RBPJ-OE Mφ-Exos inhibit tumor growth by LBX1-AS1/miR-182-5p/FOXO3 pathway in mouse xenograft tumor model. **(A)** The level of LBX1-AS1 was detected by qRT-PCR. **(B)** The level of miR-182-5p was detected by qRT-PCR. **(C)** The level of FOXO3 was detected by qRT-PCR. **(D)** Representative images of xenograft tumors (three mice per group) in nude mice. **(E)** Tumor volume is monitored every 7 days for 35 days. **(F)** The weights of xenograft tumors are summarized. *P < 0.05, **P < 0.01 and ***P < 0.001 for statistical differences.

For proving the effect of Mφ-Exo-*LBX1-AS1* on the modulation of OSCC growth *in vivo*, SCC-4 cells transfected with sh-lncRNA or sh-NC or cocultured with WT Mφ-Exos, *RBPJ-OE* Mφ-Exos, or *RBPJ-OE* Mφ-Exo-sh-lncRNA were subcutaneously injected into nude mice. The level of LBX1-AS1, miR-182-5p, and FOXO3 from the xenograft tumors were detected by qRT-PCR (**Figures 8A–C**). Tumors cultured with *RBPJ-OE* Mφ-Exos were significantly smaller, whereas those undergoing sh-lncRNA transfection were significantly larger. The greatest differences in the tumor volume and weight were observed in the tumors between *RBPJ-OE* Mφ-Exos group and sh-lncRNA group (**Figures 8D–F**). What’s more, the inhibitory effects of *RBPJ-OE* Mφ-Exos on the tumor growth *in vivo* were eliminated when LBX1-AS1 was knocked down in Exos (**Figures 8D–F**). These results signify that *RBPJ-OE* Mφ-Exos might inhibit tumor growth through a *LBX1-AS1*/miR-182-5p/*FOXO3* pathway in xenograft tumor models.

## Discussion

Macrophages are abundant in the OSCC tumoral environment and associated with its progression (31, 32). Moreover, the macrophage environment is heterogenous with the progression of tumors dependent on alternatively polarized M2 macrophages and tumorigenic immune responses dependent on M1-polarized macrophages (33, 34). Therefore, improving the understanding of macrophage regulation in the tumoral environment is important in developing effective therapies for OSCC. Notch-*RBPJ* signaling is believed to regulate TLR-induced inflammatory macrophage polarization by the indirect regulation of M1-specific genes (24).

In this study, we examined whether overexpressing *RBPJ* in macrophages would influence OSCC cells. We found that *RBPJ-OE* Mφ-Exos could inhibit cell proliferation and invasion of OSCC cells. Furthermore, we probed their interrelations by investigating the differentially regulated lncRNAs in Mφ-Exos with upregulated *RBPJ*. Using the lncRNA microarray technique, we discovered that 27 Exo-lncRNAs were differentially regulated in WT Mφ-Exos with *RBPJ* overexpression, of which 24 were upregulated and 3 were downregulated. Later, we selected highest expressed lncRNA, *LBX1-AS1* for further analysis. Then we unveiled that the inhibitory effects of *RBPJ-OE* Mφ-Exos on the proliferation and invasion of OSCC cells (SCC-4 and CAL-27) were eliminated when *LBX1-AS1* was knocked down in Exos. Meanwhile, *LBX1-AS1* overexpression dramatically repressed OSCC cells to proliferate and invade. These associations required further investigation, so we searched for miRNAs that may interact with *LBX1-AS1*.

The public database (starBase) predicted that *LBX1-AS1* may interact with miR-182-5p, which was validated *via* dual luciferase reporter and RIP assays. Our studies proved that LBX1-AS1 and miR-182-5p constitute the same RISC. Then, a negative correlation between miR-182-5p and *LBX1-AS1* in OSCC and matched para-carcinoma tissues was confirmed by Pearson’s analysis. Thus, we deduced that *LBX1-AS1* may repress miR-182-5p to prevent it from interacting in other pathways. StarBase revealed that miR-182-5p interacted with *FOXO3*, a well-known tumor suppressor gene (27–29). Furthermore, FOXO3a reactivation mediates the synergistic cytotoxic effects of rapamycin and cisplatin in oral squamous cell carcinoma cells (35). In the current research, we found that *LBX1-AS1* overexpression upregulated *FOXO3* and inhibited cells to proliferate and invade whereas miR-182-5p mimics transfection reversed them in OSCC cells. What’s more, *FOXO3* expression displayed a negative interrelation with miR-182-5p level and a positive correlation with *LBX1-AS1* level in OSCC tissues. *In vivo* assays further verified that *RBPJ-OE* Mφ-Exos might inhibit tumor growth through a *LBX1-AS1*/miR-182-5p/*FOXO3* pathway in xenograft tumor models.

This research has several deficiencies. First, the effect of the exosomes derived from LBX1-AS1-overexpressed macrophage cells on OSCC cells should be explored. Second, it is worth trying to inject RBPJ-overexpressed macrophage-derived exosomes daily into OSCC-injected mice to examine its therapeutic potential. Third, another macrophage activation method (non-transgenic) should be included in this study to further confirm the effects of activated macrophage-derived exosomes.

To conclude, *LBX1-AS1* suppress OSCC cells to proliferate and invade *via* the miR-182-5p/*FOXO3* pathway. Moreover, *RBPJ-OE* Mφ-Exos inhibits tumor growth by stimulating the *LBX1-AS1*/miR-182-5p/*FOXO3* pathway *in vitro* and *in vivo*. The above results indicate that *RBPJ-OE* Mφ-Exos probably play a potential regulation role in the OSCC progression and *LBX1-AS1* could be a biomarker for OSCC diagnosis and potential target for OSCC therapy.

## Data Availability Statement

The data presented in the study are deposited in the ArrayExpress repository, accession number (E-MTAB-9989).

## Ethics Statement

The studies involving human participants were reviewed and approved by Foshan Stomatological Hospital, School of Stomatology and Medicine, Foshan University (FSU2016033). The patients/participants provided their written informed consent to participate in this study. The animal study was reviewed and approved by Foshan Stomatological Hospital, School of Stomatology and Medicine, Foshan University.

## Author Contributions

CZ and XL designed the study. YA, CZ, and ZT performed the experiments. HW and SW collected and analyzed the data. CZ was a major contributor in developing the first draft of this manuscript. CZ and XL revised this manuscript. All authors contributed to the article and approved the submitted version.

## Conflict of Interest

The authors declare that the research was conducted in the absence of any commercial or financial relationships that could be construed as a potential conflict of interest.

## Supplementary Material

Supplementary Figure 1 RBPJ-OE Mφ-Exos can’t influence the expression of RBPJ in OSCC cells. **(A)** The relative expressions of RBPJ in WT Mφ and RBPJ-OE Mφ were detected by qRT-PCR. **(B)** The relative expressions of RBPJ in WT Mφ-Exos treated SCC-4/CAL-27 cells and RBPJ-OE Mφ-Exos treated SCC-4/CAL-27 cells were detected by qRT-PCR. ***P < 0.001, ns: no significance.
