# Supplementary figures and images for: Exosomal LncRNA LBX1-AS1 Derived From RBPJ Overexpressed-Macrophages Inhibits Oral Squamous Cell Carcinoma Progress via miR-182-5p/FOXO3
Source: Front Oncol. 2021 Mar 17;11:605884. doi: 10.3389/fonc.2021.605884 (PMC8010199; doi:10.3389/fonc.2021.605884)

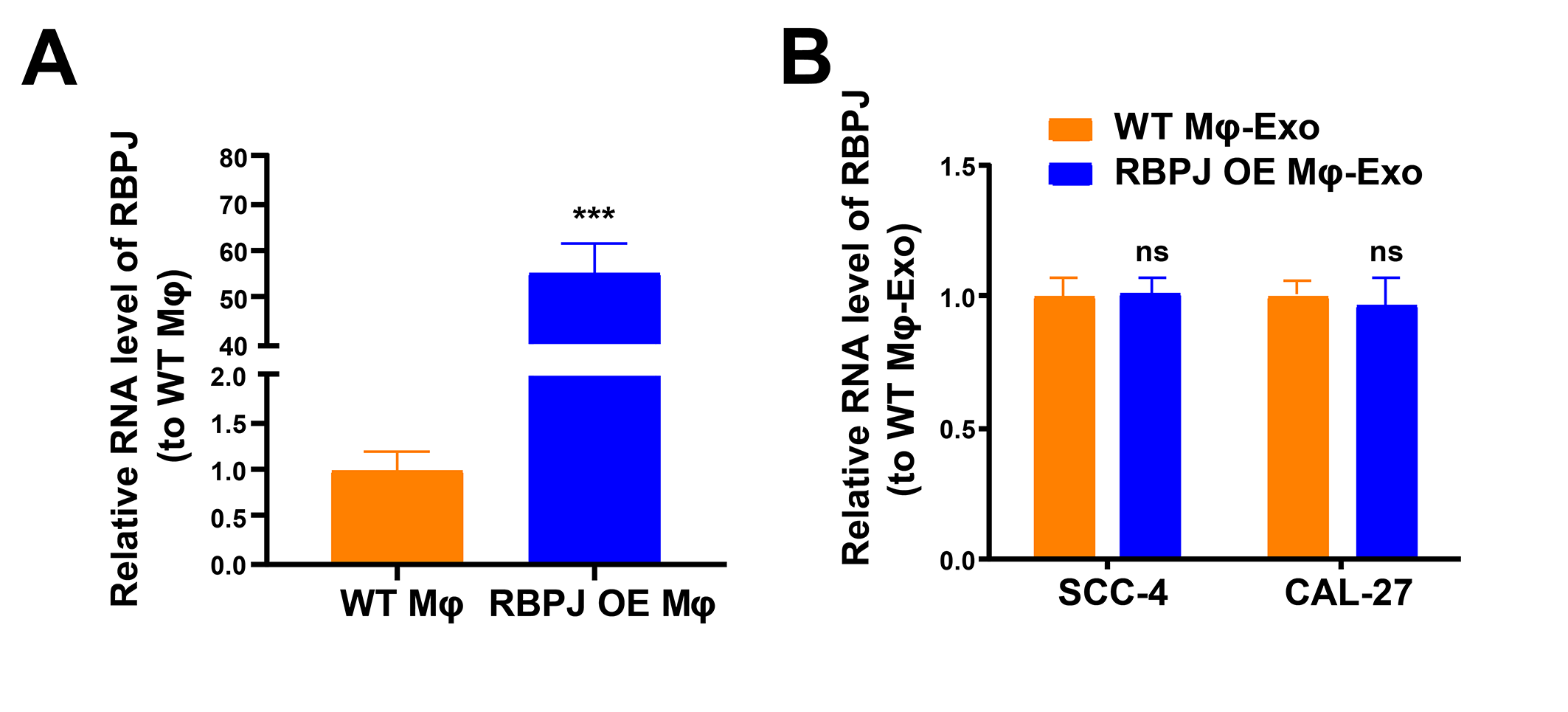

Supplement: Supplementary Figure 1 — RBPJ-OE Mφ-Exos can’t influence the expression of RBPJ in OSCC cells. (A) The relative expressions of RBPJ in WT Mφ and RBPJ-OE Mφ were detected by qRT-PCR. (B) The relative expressions of RBPJ in WT Mφ-Exos treated SCC-4/CAL-27 cells and RBPJ-OE Mφ-Exos treated SCC-4/CAL-27 cells were detected by qRT-PCR. ***P < 0.001, ns: no significance. [file Image_1.tif]
